# Relevance of Chronic Lyme Disease to Family Medicine as a Complex Multidimensional Chronic Disease Construct: A Systematic Review

**DOI:** 10.1155/2014/138016

**Published:** 2014-11-24

**Authors:** Liesbeth Borgermans, Geert Goderis, Jan Vandevoorde, Dirk Devroey

**Affiliations:** ^1^Department of Family Medicine & Chronic Care, Vrije Universiteit Brussel (VUB), Laarbeeklaan 103, 1090 Brussels, Belgium; ^2^Department of General Practice and University Hospitals Leuven, Katholieke Universiteit Leuven (KUL), Kapucijnenvoer 33, 3000 Leuven, Belgium

## Abstract

Lyme disease has become a global public health problem and a prototype of an emerging infection. Both treatment-refractory infection and symptoms that are related to *Borrelia burgdorferi* infection remain subject to controversy. Because of the absence of solid evidence on prevalence, causes, diagnostic criteria, tools and treatment options, the role of autoimmunity to residual or persisting antigens, and the role of a toxin or other bacterial-associated products that are responsible for the symptoms and signs, chronic Lyme disease (CLD) remains a relatively poorly understood chronic disease construct. The role and performance of family medicine in the detection, integrative treatment, and follow-up of CLD are not well studied either. The purpose of this paper is to describe insights into the complexity of CLD as a multidimensional chronic disease construct and its relevance to family medicine by means of a systematic literature review.

## 1. Introduction

Lyme disease is a worldwide-distributed multisystem animal-borne disease, caused by* Borrelia burgdorferi* (Bb) sensu lato (Gram-negative Spirochaetes) which is a group of at least 12 closely related species [1]. Recent evidence shows that human cases of Lyme disease may also be caused by more than one species of* B. burgdorferi* sensu lato [2], with rodents and small mammals as its animal reservoir [3]. Traditionally Lyme disease is divided into four stages, and clinical manifestations may be both cutaneous and systemic and can have cardiovascular, neurological, and musculoskeletal involvement [4–6]. When Lyme disease is treated with appropriate antibiotic therapy in the early stages, long-term outcomes are good [7].

Lyme disease is the most common vector-borne disease in the USA and Europe with more than 300,000 new cases in the USA [8] and 100.000 cases annually in Europe (estimated from available national data) [9]. However, this number is largely underestimated as case reporting is highly inconsistent and many infections go undiagnosed [10–13]. Global climate change expanding the range of tick vectors and an increase in the incidence suggest that this disease will remain an important health issue in the forthcoming decades [14].

### 1.1. Definition of Chronic Lyme Disease (CLD)

Chronic Lyme disease (CLD) is considered a constellation of persistent symptoms in patients with or without evidence of Bb infection [15, 16]. Some authors use the term CLD only for patients who had been clinically diagnosed with Lyme disease and have persisting symptoms lasting more than 6 months following antibiotic treatment [8]. For this reason the term “posttreatment Lyme disease” [17, 18] or “post-Lyme disease syndrome” [10, 18] is often used. The category of “probable Lyme disease” was recently added to the CDC surveillance case definition to describe patients with serologic evidence of exposure and physician-diagnosed disease in the absence of objective signs [19]. Recently, the term chronic arthropod-borne neuropathy (CAN) has been proposed as an alternative to chronic Lyme disease, as calling the chronic illness “Lyme disease” causes confusion to patients and physicians [20].

### 1.2. Evidence on Persistent Bb Infection

There is growing and well-documented evidence to the concept of persistent Bb infection in both animals [21–31] and humans [32–41]. Recent evidence shows Bb is able to escape from destruction by the host immune reactions, persist in host tissues, and sustain chronic infection and inflammation, despite aggressive antibiotic challenge [32, 35, 36, 42–44]. An estimated 20% of patients display recurrent symptoms after antibiotic treatment [45]. A recent study showed that, at six months following antibiotic treatment, 36% of patients reported new-onset fatigue, 20% widespread pain, and 45% neurocognitive difficulties without evidence of depressive symptomatology [46]. Some studies have provided evidence on pleocytosis and the production of Bb antibodies in the cerebrospinal fluid of patients with neuroborreliosis [47], while this is contradicted by other studies showing greater variability in such patients [48]. When looking at the clinical and basic science research, it is apparent that persistent Bb infection and associated inflammation and molecular mimicry mechanisms are associated with gradually increasing encephalopathy and gradually increasing mental symptoms [49]. Despite available evidence, both treatment-refractory infection and persistent symptoms that are related to Bb infection remain subject to controversy in which national medical societies, patient advocacy groups, insurance companies, lawyers, doctors, the private health medical sector, and scientific journals all have become embroiled [10, 50–53]. The controversy is exacerbated by the lack of a clinical or microbiologic definition and the frequent occurrence of chronic symptoms in the general population [54]. The Lyme disease controversy can also be largely linked to the misconception that neurobehavioral effects of illness constitute evidence of nervous system infection. Appropriate differentiation between neuroborreliosis (nervous system* Borrelia burgdorferi* infection) and Lyme encephalopathy (altered nervous system function in individuals with systemic but not nervous system infection)—or encephalopathies of other etiologies—would lessen the controversy considerably, as the attribution of nonspecific symptoms to supposed ongoing central nervous system infection is a major factor perpetuating the debate [17].

### 1.3. CLD as a Poorly Understood Chronic Disease Construct

Because of the absence of solid evidence on prevalence [20], causes [55], diagnostic criteria and treatment options [56], the role of autoimmunity to residual or persisting antigens, and the role of a toxin or other bacterial-associated products that are responsible for the symptoms and signs [57], CLD remains a relatively poorly understood chronic disease construct. The role and performance of family medicine in the detection, integrative treatment, and follow-up of CLD are not well studied either. The purpose of this paper is to describe the complexity of CLD as a multidimensional chronic disease construct and its relevance to family medicine by means of a systematic literature review. This paper is a second paper in a series on chronic diseases [58] that focus on complex chronic conditions.

## 2. Methods

A systematic review method was used to document the complexity and multidimensionality of CLD. In addressing the objective of this review, we used a parallel search strategy via Medline, The Cochrane Library of Systematic Reviews, CINAHL (Cumulative Index to Nursing and Allied Health Literature), and PRE-CINAHL, complemented with a reference review of key articles. Articles had to be published between October 2009 and October 2014.

Articles were selected if they dealt with (1) CLD care complexity; (2) CLD case (patient) complexity; (3) the complexity of CLD quality assessment; and (4) complexity of CLD at the health system level. In addition the studies had to produce insights into the complexity of CLD with relevance to family medicine. Nonsystematic reviews, opinions, and grey literature were excluded from the search. The search strategy included a combination of Medical Subject Headings (MeSH) terms with regard to Lyme disease. We also scrutinised extra sources for further identification of studies by hand-searching the reference lists of all articles. By focusing on CLD care, case, quality assessment, and health systems complexity, we further build on four major and interrelated components of complexity in chronic care that have been described by Borgermans et al. [59]. Each of these components represents a range of elements that contribute to the picture of complexity in chronic care.

Two authors carried out independent screening of titles and abstracts using the specific inclusion and exclusion criterion reported on previously. We ordered the full text of all citations that met the eligibility criteria or appeared relevant or where relevance/eligibility was not clear from the abstract. In the final screening, two authors independently scrutinised the full texts of studies and recorded the reasons when articles were excluded resolving disagreements by discussion and when necessary referred to a third author. A tool-based assessment of the methodological quality of the studies was not part of this review.

**Figure 1:**
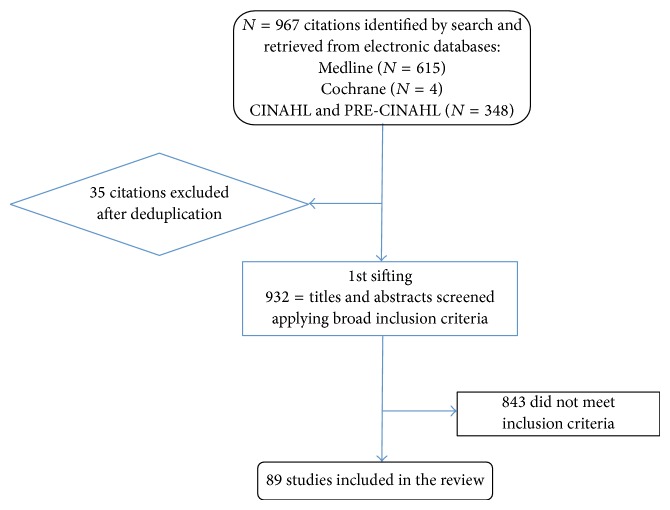
Flow diagram of study selection process.

The flow diagram (Figure 1) shows our study selection process and the number of studies included.

## 3. Results

A total of 967 studies were identified. Finally 945 articles were included for assessment. 72 studies provided insights on CLD care complexity, 9 studies provided insights on CLD case complexity, 2 studies provided insights on CLD quality complexity, and 6 studies provided insights on CLD health system complexity with relevance to family medicine.

### 3.1. Care Complexity

#### 3.1.1. Diagnosis

It is current practice for physicians to base themselves primarily on clinical signs and symptoms when CLD is suspected. Patients often cannot recall being bitten by a tick or if erythema migrans has occurred. Erythema migrans is pathognomonic and does not require any further laboratory investigations [60], but a careful analysis is recommended as a variety of unconventional histopathologic patterns may occur [61]. In the case of no erythema migrans clinical manifestations are complemented by laboratory tests, including enzyme-linked immunosorbent assay (ELISA) and Western blot testing [62]. Although this two-tier testing is standard practice in both the United States and Europe, the test kits generally differ [63].

There is consistent evidence that the two-tier testing lacks sensitivity, cannot distinguish between current and past infection, cannot be used as a marker for treatment, is often dependent on subjectively scored immunoblots, and is considered expensive [64, 65]. Moreover, the diagnosis of CLD based on clinical manifestations, serological findings, and detection of infectious agents often contradict each other [56, 66, 67]. For this reason a growing number of studies support two-tier testing followed by a sensitive and reliable DNA sequencing for confirmation to support the diagnosis of CLD [68, 69]. Other authors suggest the use of a single tier hybrid antigen immuno-PCR and detection of IgG antibodies only [64]. For cases with a high clinical suspicion of disease, the C6 peptide enzyme immunoassay (EIA) is put forward as a stand-alone test or in the second tier of a 2-tiered algorithm [70]. This assay is based on a peptide (C6), whose amino acid sequence reproduces a conserved, immunodominant region of a single protein of* Borrelia burgdorferi*. Overall, the use of PCR-based methods appears to be of importance because of the high sensitivity and specificity of these assays [71–73]. However, PCR positivity in the absence of culture positivity following antibiotic treatment should be interpreted with caution since* B. burgdorferi* DNA and mRNA can be detected in samples long after spirochetes are no longer viable as assessed by classic microbiological parameters [74].

Another novel method including culturing spirochetes from the serum of patients uses a two-step preenrichment process, followed by immunostaining with or without polymerase chain reaction (PCR) analysis [75]. In patients who complied with the strict CDC surveillance case definition for Lyme disease the procedure resulted in positive cultures in 47% at 6 days and 94% at week 16. The Centers for Disease Control and Prevention has however raised concerns about false-positive results caused by laboratory contamination and the potential for misdiagnosis [76]. Some authors put forward the measurement of activity of two lysosomal exoglycosidases, *α*-fucosidase (FUC) and *β*-galactosidase (GAL), in the serum as potential markers of LD [77].

Recent evidence has shown that the presence or absence of chronic Lyme borreliosis may be objectively adjudicated by tissue examinations which demonstrate or which fail to show pathogenic microbes in patients who have received a full course of antibiotics [78]. The use of SPECT scans of the brain using Tc and standard nuclear imaging techniques is considered an objective measure of abnormalities present in patients with otherwise difficult to objectify clinical findings [79]. Brain SPECT scans are abnormal in most patients with chronic Lyme disease, and these scans can be used to provide objective evidence in support of the clinical diagnosis [79].

Overall, studies highlight the need for standardization in diagnostic (serological) testing, as well as the need for studies that discriminate between active disease and past infection.

#### 3.1.2. Differential Diagnosis

A factor that complicates the diagnosis of CLD is that it does not present with isolated subjective symptoms [47]. In undiagnosed patients or posttreatment, Bb may mimic the symptoms and signs of other diseases disorders [80, 81] and for this reason is called “the illness with a thousand faces.” Patients present themselves with a wide variety of (often unrelated) clinical manifestations [82]. These symptoms may include fatigue [83], insomnia [49], widespread pain [19], arthritis [84], chronic eye lid edema [85], cataract [86], dermatitis [87], cognitive complaints [19], bilateral hearing loss [88], adult respiratory distress syndrome [89], palpitations, tachycardia [90], syncope [87], tremors, epileptic crises [91], depression, anxiety, panic attacks, catatonia, and psychosis [92], amongst others [93, 94]. Lyme disease might become misdiagnosed as fibromyalgia [95], chronic fatigue syndrome [96, 97], amyotrophic lateral sclerosis [80], autism spectrum disorder [98], Parkinson's disease, rheumatoid arthritis [99], multiple chemical sensitivity, Guillain-Barre syndrome (GBS) [80], and dementia [100], as well as other numerous neurological [101, 102] and psychiatric disorders [88, 103]. Caution is however warranted as misdiagnosis of CLD may result in unnecessary antibiotic courses [104].

Overall, studies show the need for a careful differential diagnosis in patients with suspected CLD and persistent complaints.

#### 3.1.3. Diagnosis of Coinfections

The diagnosis of CLD is even more complex when tick-borne coinfections occur in association with Lyme disease [105], which is the rule rather than the exception [106]. Routine laboratory tests exhibit varying reported sensitivity and are usually unhelpful in diagnosis, as serology fails in terms of specificity and/or sensitivity [107]. Clinically relevant coinfections include* Bartonella* species [105],* Babesia* [108],* Anaplasma* [109],* Rickettsia* [110],* Yersinia enterocolitica*,* Chlamydophila pneumoniae*,* Chlamydia trachomatis*,* Mycoplasma pneumoniae* [105], and tick-borne encephalitis virus, amongst others [108, 111]. A particular condition that gets growing attention in patients with CLD is Morgellons disease. Morgellons disease is an emerging skin disease characterized by formation of dermal filaments associated with multisystemic symptoms [112]. Recent studies show that Bb spirochetes were detected in the dermatological specimens from study patients, providing evidence that Morgellons disease is associated with an infectious process [112–115].

Overall, studies highlight the importance of coinfections since they can complicate the diagnostic process and their pathological synergism can exacerbate CLD or induce similar disease manifestations.

#### 3.1.4. Treatment

Treatment options are complicated since the population of individuals reporting CLD are heterogeneous with guidelines contradicting each other. Recommendations about the type and duration of treatments in patients with CLD have no factual basis [57], although prolonged courses of antibiotics are likely to be helpful [52]. Recent biostatistical review reveals that retreatment has meaningful clinical benefit in patients who had prior antibiotic treatment [116, 117], with the use of longer (parenteral) antibiotic therapy often to be justified [118]. Doxycycline is often the preferred agent for oral treatment because of its activity against other tick-borne illnesses, but recent evidence shows that doxycycline-treatment only does not always lead to clinical improvements in either the persistent symptoms or quality of life in patients with CLD [55]. This finding can potentially be explained since single antibiotic use often fails to address the different morphological forms of Bb as well as biofilm formations in patients with CLD. Studies indicate the need to include cell wall and cystic and intracellular drugs in any treatment as the different morphological forms of Bb display differences in sensitivity to antibiotic treatment [119]. As coinfections are present in most patients, the use of specific antibiotics is required. Tetracyclines and macrolides are often used, with quinolones for alternative treatment, particularly gemifloxacin. For* Bartonella henselae*,* Chlamydia trachomatis*, and* Chlamydophila pneumoniae* the combination with rifampicin is recommended [105].

Recent evidence shows that novel therapeutic targets for the treatment of the disease should acknowledge a central role of the neutrophil-activating protein A (NapA) of Bb in promoting both regulatory T-cell response and immune suppression in the cerebrospinal fluid of patients with chronic Lyme borreliosis [120].

Overall, studies show that various therapeutic regimens are used in patients with CLD reflecting the need for individualized approaches.

### 3.2. Case Complexity

The vector model of complexity (VMC) [121] is a useful model to describe case complexity in patients with CLD. The vector model proposes that the complexity of an individual patient arises out of interactions between different domains: biology, genetics, socioeconomics, environment, behaviour, culture, and the health system. In a chronic condition such as CLD, these “forces” are not easily discerned.

#### 3.2.1. Biology/Genetics

Complexity in CLD along the biology/genetics axis is important since gender distribution in patients with Lyme borreliosis has recently been demonstrated. Patients with cutaneous manifestations of Lyme borreliosis tend to be predominantly female, whereas those with noncutaneous manifestations are predominantly male [122].

#### 3.2.2. Socioeconomics/Environment

Complexity in CLD is introduced along the environment axis with a growing number of studies to document the important relationship between the increase in outdoor activities in wooded areas and the incidence of Lyme disease [123]. Special environmental risk factors include the presence of deer ticks in the home environment, ground cover containing moist humus, and leaf litter in the yard. Distribution areas as well as host and vector ranges of Lyme borreliosis agents turned out to be much wider than previously thought [2, 124]. Another element from the environment that contributes to the complexity of CLD is that the variety of Bb genospecies leads to distinction in clinical manifestations of Lyme borreliosis (LB) [125, 126]. At present, the risk of exposure to multiple pathogens from a single tick bite and the sources of coinfected ticks are not well understood [108].

#### 3.2.3. Behavior

Complexity in CLD is also introduced along the behavioral axis as CLD has considerable implications on daily life. Patients report a significant and severe decline in health status associated with chronic Lyme disease [15]. The psychological factors in the prediction of Lyme disease course have been poorly studied. Studies have shown that a history of severe, long-term, premorbid, and psychological stress is associated with increased incidence of chronic physical symptoms in Lyme disease patients. Traumatic psychological experiences predating onset of Lyme disease symptoms may play an important etiologic role in the chronicity of these symptoms [127]. As a consequence, the burden of CLD is likely to be underestimated as a consequence of the inadequate recognition of the connection between mental and physical health.

### 3.3. Quality Assessment Complexity

The complexity of quality assessment is reflected by the lack of tools at the present time that can assess the quality of care delivered to patients with CLD. A limited number of quality indicators at the structure, process, or outcome level of care for patients with CLD exist [8], which is in part a consequence of the lack of research on (the construct of) CLD. In addition, publications that describe approaches to patient involvement in quality indicator development are scarce [128] and to our knowledge nonexisting for patients with CLD.

### 3.4. Health System Complexity

Health system complexity is of relevance to health seeking behaviour of patients with CLD. There is an abundance of studies on health seeking behaviour highlighting the importance of health system characteristics and their influence on an individual's behaviour at a given time and place [129, 130]. These influences include the financing of care, access to care, coordination mechanisms, and existing stigma on unexplained medical conditions and values and norms. A large survey conducted in the USA in patients with CLD demonstrated extensive delays in obtaining an initial diagnosis and having poor access to healthcare [46, 131]. Patients with CLD are often unsatisfied with care in conventional settings [15]. Negative experiences are associated with reports of dismissive, patronizing, and condescending attitudes in health care providers. Studies show that consultations with complementary and alternative medicine (CAM) practitioners and use of CAM therapies are common [132], representing a cost to patients to be exorbitant and prohibitive.

#### 3.4.1. Relevance of CLD as a Multidimensional Chronic Disease Construct to Family Medicine

We have outlined the importance of case, care, quality assessment, and health system complexity in patients with CLD. The majority of studies focus on CLD care and case complexity with a minority of studies to report on CLD quality assessment and health system complexity.

While specialists are an essential element of the total health care continuum, the majority of patients with CLD will continue to access the health care system through family physicians [133], with hospitalization due to Lyme disease to be rare [134]. But there may be discrepancies in disease awareness among family physicians [135]. A range of persistent misconceptions in family medicine exists, ranging from the reliability of available diagnostic tools, the signs and symptoms of nervous system involvement, the importance of coinfections, the appropriate choice and duration of antimicrobial therapy, the importance of Jarisch-Herxheimer reaction after the commencement of treatment with antibiotics, the curability of the infection, and the cause of symptoms that may persist in some patients after treatment [136–138]. Lyme literate family physicians seem to be rare which provides argument to the diagnosis and treatment of CLD within the context of a broad-based, multidisciplinary approach to determining the best approach [139]. Our analysis on complexity in CLD highlights the importance of comprehensiveness in this disorder. Comprehensiveness is the ability of the family physician to address a broad range of patient problems, whether or not the conditions are within the traditional domain of the specialty in which the physician is trained. Referral to Lyme literate specialists is essential both to meet the patient's needs and to establish an integrated care network where responsibilities and tasks are shared. An effective response to the health needs of those with CLD will especially require family physicians and specialists to expand their collaborative efforts and knowledge of each other's practices and treatments. Collaboration between family physicians, dieticians, and physiotherapists is considered important since healthy lifestyles are essential in the recovery of patients with CLD [140].

Another component of the care responsibility of family physicians is ensuring that patients receive preventive interventions. Prevention of mortality and morbidity may depend on correct early diagnosis and treatment [141]. Family physicians can contribute to public health interventions in high-incidence age groups focusing on accurately communicating risk, improving knowledge both of Lyme disease symptoms and of ticks that carry the disease, as well as teaching preventive behaviors to help reducing tick-borne illness rates [142]. Research has shown that patients' knowledge of tick-borne illnesses is poor [143] and the frequency of practicing preventive behaviors is low [142]. It must be noted, however, that a growing number of patients consult medical sources on the Internet with the aim of increasing their health literacy on the subject. Family physicians must encourage and support their patients in search of reliable sources of information.

## 4. Conclusion

The controversy on CLD needs to be solved because of the heavy burden of illness associated with CLD in patients with or without evidence of Bb infection. There is a need for the development and establishment of new clinical diagnostic tools with increased accuracy, sensitivity, and specificity, as well as novel treatment approaches that may reduce the burden of illness and concomitant costs posed by CLD. Family physicians have a crucial role to play in the prevention and treatment of the disease fostering an integrative multidisciplinary approach to care.
